# 城市居民单羟基多环芳烃暴露特征与影响因素分析

**DOI:** 10.3724/SP.J.1123.2024.10006

**Published:** 2025-06-08

**Authors:** Hui FU, Yifu LU, Linna XIE, Ying ZHU, Zheng LI, Xiaojian HU

**Affiliations:** 中国疾病预防控制中心环境与人群健康重点实验室，中国疾病预防控制中心环境与健康相关产品安全所，北京 100021; China CDC Key Laboratory of Environment and Population Health，National Institute of Environmental Health，Chinese Center for Disease Control and Prevention，Beijing 100021，China

**Keywords:** 多环芳烃, 气相色谱-高分辨双聚焦磁质谱, 单羟基多环芳烃, 暴露特征, 影响因素, polycyclic aromatic hydrocarbons （PAHs）, gas chromatography-high resolution dual-focus magnetic mass spectrometry （GC-HRMS）, monohydroxypolycyclic aromatic hydrocarbons （OH-PAHs）, exposure characteristics, influencing factors

## Abstract

为了解城市地区非职业暴露居民多环芳烃（PAHs）暴露现状，研究采用同位素稀释结合液液萃取-气相色谱-高分辨双聚焦磁质谱技术，对北京市92名2～80岁常住居民尿液中10种单羟基多环芳烃（OH-PAHs）进行定量分析，并以尿肌酐校正OH-PAHs含量。低于检出限的结果以检出限的1/2代替。通过Spearman秩相关分析（双尾）评估OH-PAHs间的相关性，并用非参数Mann-Whitney U检验及Kruskal-Wallis H检验比较不同人群OH-PAHs含量分布。结果显示，1-羟基萘、2-羟基萘、2-羟基芴、9-羟基芴、1-羟基菲和2-羟基菲等6种OH-PAHs的检出率达到100%。10种OH-PAHs的总含量（ΣOH-PAHs）为661～33 782 ng/g，几何平均值（GM）为2 775 ng/g，个体间差异显著。OH-PAHs含量分布趋势为羟基萘>羟基芴>羟基菲>1-羟基芘，与分子大小显著负相关，羟基萘为主要化合物，占比62.2%。OH-PAHs之间存在复杂的相关性，9-羟基芴显示出独特的暴露模式。尿液中OH-PAHs含量与性别、年龄相关，而吸烟是重要的影响因素。ΣOH-PAHs在少年儿童组（0~15岁，GM为3 940 ng/g）达到峰值，劳动年龄组（16~59岁，GM为2 598 ng/g）和老年组（≥60岁，GM为2 639 ng/g）水平相近，显示年龄为关键影响因素。吸烟习惯对OH-PAHs含量有一致且显著的影响，吸烟者的OH-PAHs含量普遍高于非吸烟者。男性总体暴露水平高于女性，但排除吸烟因素后，女性1-羟基芘含量水平显著高于男性，提示性别间代谢和行为差异对PAHs暴露有影响。研究揭示了北京居民OH-PAHs的暴露情况及含量分布特征，为PAHs污染及健康影响研究、流行病学调查、疾病负担评估及政策制定提供了科学依据。

## Abstract (en)

Polycyclic aromatic hydrocarbons （PAHs） are organic pollutants that are ubiquitous in nature and in environments affected by anthropogenic activities. PAHs enter the human body through inhalation， ingestion， or skin contact， and consequently threaten human health. Urban areas are mostly affected by PAHs pollution， which is ascribable to dense population， heavy traffic， and limited air-pollutant diffusion. In this study， we assessed the current status of PAHs exposure among non-occupationally exposed urban residents using isotope dilution combined with liquid-liquid extraction-gas chromatography-high resolution dual-focus magnetic mass spectrometry （GC-HRMS）. The burden of PAHs of 92 permanent residents aged 2 to 80 years in Beijing was investigated. Ten monohydroxypolycyclic aromatic hydrocarbons （OH-PAHs） were measured in urine samples， namely hydroxynaphthalene （OHNap， including 1-OHNap and 2-OHNap）， hydroxyfluorene （OHFlu， including 2-OHFlu， 3-OHFlu， and 9-OHFlu）， hydroxyphenanthrene （OHPhe， including 1-OHPhe， 2-OHPhe， 3-OHPhe， and 4-OHPhe）， and 1-hydroxypyrene （1-OHPyr）. The OH-PAH levels were corrected with urinary creatinine， with results below LODs replaced with half the value of LODs. Correlations between OH-PAHs were assessed using Spearman’s rank correlation analysis （two-tailed）. The nonparametric Mann-Whitney U test and the Kruskal-Wallis H test were used to compare the distribution of OH-PAH levels in different populations. The results showed that six OH-PAHs （1-OHNap， 2-OHNap， 2-OHFlu， 9-OHFlu， 1-OHPhe， and 2-OHPhe） were detected in all the urine samples. The total contents （ΣOH-PAHs） of the 10 OH-PAHs ranged from 661 to 33 782 ng/g， with an overall geometric mean （GM） of 2 775 ng/g and significant inter-individual differences. The following content-distribution trend was observed： OHNap>OHFlu>OHPhe>1-OHPyr， with a significant negative correlation with molecular size recorded. OHNap was mainly observed， accounting for 62.2% of the total. Complex correlations were found to existed between the OH-PAHs， with 9-OHFlu exhibiting unique exposure patterns. Urinary OH-PAH levels were found to correlate with gender and age， and smoking was also observed to be a significant influencing factor. ΣOH-PAHs peaked in the youth group （0‒15 years）， with a GM of 3 940 ng/g. Levels of ΣOH-PAHs were similar in the working-age group （16‒59 years， GM： 2 598 ng/g） and in the elderly group （≥60 years， GM： 2 639 ng/g）. These suggest that age is a key PAH-exposure factor. Habitual smoking was found to consistently and significantly affect OH-PAH levels， with smokers generally having higher levels of OH-PAH than non-smokers. While males exhibited higher overall exposure levels than females， females exhibited significantly higher levels of 1-OHPyr than males （*p*=0.03） when smoking was excluded， which suggests that metabolic and behavioral differences between genders impact PAHs exposure. This study revealed the exposure and distribution characteristics of OH-PAHs in Beijing residents. It provides a scientific basis for studying PAHs pollution and its health effect， as well as epidemiological investigations， disease-burden assessments， and policy formulation.

多环芳烃（PAHs）是一类广泛分布于自然及人为活动环境中的有机污染物，因其对人体潜在的致癌与致突变危害而备受关注^［1，2］^。这类化合物的来源极为广泛，涵盖机动车尾气排放、住宅和工业供暖过程、煤炭及化石燃料（原油和天然气）的加工、废弃物焚烧、烟草烟雾以及多种制成品（如药品、染料、塑料和农药）的生产与使用^［3］^。人类主要通过吸入、摄入及皮肤接触等途径暴露于PAHs^［4］^，其中，非职业暴露的主要来源包括烟草烟雾吸入、食物摄入及空气污染^［5］^。进入体内的PAHs经代谢后，以代谢产物的形式通过尿液排出体外，其中羟基化的多环芳烃是主要的代谢产物之一。长期接触PAHs可显著增加皮肤、肺、胰腺、食道、膀胱、结肠和女性乳房等多个器官的肿瘤风险，并可能提高肺癌及心血管疾病（如动脉粥样硬化、血栓形成、高血压和心肌梗死）的发病率^［2］^。近年来，研究进一步揭示了PAHs暴露的深远影响，如女性产前接触会增加自发性早产或胎龄缩短的风险^［6］^。此外，流行病学证据也指出了PAHs暴露与儿童哮喘之间的关联^［7‒10］^。随着全球城市化进程的加速，城市区域因人口密集、交通繁忙及大气污染物扩散受限，已成为PAHs污染的高风险区域，人类健康面临严峻挑战^［2，3］^。

北京作为一座人口密集、交通高度繁忙的大都市，其居民面临的PAHs暴露风险尤为突出^［11］^。尿液中的单羟基多环芳烃（OH-PAHs）作为PAHs的暴露生物标志物，其水平反映了人体内PAHs的暴露情况^［4，12］^。本研究通过液液萃取-气相色谱-高分辨双聚焦磁质谱技术（LLE-GC-HRMS），测定了北京居民体内10种关键OH-PAHs的含量，旨在揭示该区域人群PAHs的暴露特征，为后续流行病学研究提供基础数据，为相关疾病负担评估和政策制定提供依据。

## 1 实验部分

### 1.1 仪器、试剂与材料

7890A气相色谱仪（美国Agilent公司），高分辨双聚焦磁质谱仪（EI源，美国Waters Micromass公司）。

10种OH-PAHs标准溶液：1-羟基萘（1-OHNap，纯度100%）、1-羟基芘（1-OHPyr，纯度100%）购自美国AccuStandard公司，2-羟基萘（2-OHNap，纯度99.9%）、1-羟基菲（1-OHPhe，纯度99.0%）、3-羟基菲（3-OHPhe，纯度99.5%）、4-羟基菲（4-OHPhe，纯度99.0%）购自德国Dr. Ehrenstorfer公司，2-羟基菲（2-OHPhe，纯度94.0%）购自英国LGC公司，2-羟基芴（2-OHFlu，纯度98%）、9-羟基芴（9-OHFlu，纯度98%）购自加拿大Toronto Research Chemicals公司，3-羟基芴（3-OHFlu，纯度≥98%）购自美国Cambridge Isotope Laboratories公司。同位素内标：^13^C_6_-1-OHNap、^13^C_6_-2-OHNap、^13^C_6_-9-OHFlu、^13^C_6_-3-OHPhe（质量浓度均为50 μg/mL）购自美国Cambridge Isotope Laboratories公司，D_9_-2-OHFlu、D_9_-3-OHFlu、D_9_-1-OHPhe、D_9_-2-OHPhe、D_9_-4-OHPhe、D_9_-1-OHPyr（纯度>98%）购自加拿大Toronto Research Chemicals公司。

正戊烷（GC级）购自美国Mreda公司，甲苯（HPLC级）购自美国Tedia公司，十二烷（纯度99%）购自比利时Acros公司，乙酸（优级纯）、无水乙酸钠（分析纯）、硝酸银（分析纯）购自国药集团化学试剂有限公司，L-（+）抗坏血酸（纯度99.0%）购自德国CNW公司，*β*-葡萄糖苷酸酶/硫酸芳酯酶混合酶（*β*-葡萄糖苷酸酶30 U/mL、硫酸芳酯酶60 U/mL）、*N*-甲基-*N*-（三甲基硅烷基）三氟乙酰胺（MSTFA，纯度≥98.5%）购自德国Merck公司，^13^C_12_-2，3，3‍′，4，4′-五氯联苯（^13^C_12_-PCB-105，40 μg/mL）购自美国Cambridge Isotope Laboratories公司，标准参考物质（NIST SRM 3672）购自美国NIST标准物质中心。

### 1.2 样品的采集和制备

随机招募年龄2～80岁的北京市常住居民，在家中用螺帽密封聚丙烯瓶采集空腹晨尿（30 mL），保存于冰盒中，送至实验室，5 mL冻存管分装后，‒80 ℃保存直至分析。所有参与者或其监护人签署知情同意书，并配合完成生物样本的采集及相应的问卷调查。所有问卷信息不完整或尿样不合格的样本均被排除，最终，共有92名受试者的尿液样本被纳入分析，调查对象的人口学特征见表1。该研究获得了北京市疾病预防控制中心伦理委员会的批准（编号202205）。

**表 1:** 调查对象的人口学特征

Characteristic	N （%）^*^
Gender	
Female	40 （43.5）
Male	52 （56.5）
Age （years）	
0‒15	14 （15.2）
16‒59	64 （69.6）
≥60	14 （15.2）
Smoking status	
Smoker	9 （9.8）
Non-smoker	83 （90.2）

* Numbers of respondents （percent in all， %）.

### 1.3 样品预处理及仪器分析

样品分析方法在前期研究^［13］^基础上对目标分析物的种类及质谱参数进行了优化。

尿液样本于4 ℃下解冻，平衡至室温后，取2 mL于玻璃离心管中，依次加入1 mL乙酸钠溶液（1 mol/L，pH=5.5）、20 μL抗坏血酸溶液（250 mg/mL）、20 μL混合内标溶液（200 ng/mL甲苯溶液）和50 μL *β*-葡萄糖苷酸酶/硫酸芳酯酶混合酶，混匀，经37 ℃水浴避光酶解17 h后，加入2 mL去离子水，用10 mL戊烷-甲苯（4∶1， v/v）分两次提取，每次5 mL，收集上层有机相提取液，用1 mL硝酸银溶液（1 mol/L）沉淀蛋白质。上清提取液中加入20 μL十二烷后于40 ℃、5 Pa氮吹20 min，再升温至70 ℃浓缩近干后，用20 μL甲苯复溶，转移至已加入20 μL回收率内标^13^C_12_-PCB-105（100 ng/mL甲苯溶液）和100 μL衍生化试剂MSTFA的棕色进样小瓶中，60 ℃衍生化50 min后，采用气相色谱-高分辨双聚焦磁质谱仪进行仪器分析。其中，10种OH-PAHs及同位素内标、回收率内标的保留时间、质谱参数及检出限见表2。

**表 2:** 10种OH-PAHs及同位素内标、回收率内标的保留时间、质谱参数及检出限

Analyte	Abbreviation	Molecular ion ［M^+^］ （*m/z*）	Retention time/min	Internal standard	LOD/（ng/L）
1-Hydroxynaphthalene	1-OHNap	216.0970	8.98	^13^C_6_-1-OHNap	25
2-Hydroxynaphthalene	2-OHNap	216.0970	9.23	^13^C_6_-2-OHNap	25
2-Hydroxyfluorene	2-OHFlu	254.1127	13.32	D_9_-2-OHFlu	5
3-Hydroxyfluorene	3-OHFlu	254.1127	13.11	D_9_-3-OHFlu	5
9-Hydroxyfluorene	9-OHFlu	254.1127	11.62	^13^C_6_-9-OHFlu	5
1-Hydroxyphenanthrene	1-OHPhe	266.1127	15.13	D_9_-1-OHPhe	5
2-Hydroxyphenanthrene	2-OHPhe	266.1127	15.55	D_9_-2-OHPhe	5
3-Hydroxyphenanthrene	3-OHPhe	266.1127	15.05	^13^C_6_-3-OHPhe	5
4-Hydroxyphenanthrene	4-OHPhe	266.1127	14.23	D_9_-4-OHPhe	5
1-Hydroxypyrene	1-OHPyr	290.1127	20.11	D_9_-1-OHPyr	5
^13^C_6_-1-Hydroxynaphthalene	^13^C_6_-1-OHNap	222.1172	8.98	/	/
^13^C_6_-2-Hydroxynaphthalene	^13^C_6_-2-OHNap	222.1172	9.23	/	/
D_9_-2-Hydroxyfluorene	D_9_-2-OHFlu	263.1692	13.26	/	/
D_9_-3-Hydroxyfluorene	D_9_-3-OHFlu	263.1692	13.07	/	/
^13^C_6_-9-Hydroxyfluorene	^13^C_6_-9-OHFlu	260.1127	11.62	/	/
D_9_-1-Hydroxyphenanthrene	D_9_-1-OHPhe	275.1692	15.07	/	/
D_9_-2-Hydroxyphenanthrene	D_9_-2-OHPhe	275.1692	15.49	/	/
^13^C_6_-3-Hydroxyphenanthrene	^13^C_6_-3-OHPhe	272.1127	15.07	/	/
D_9_-4-Hydroxyphenanthrene	D_9_-4-OHPhe	275.1692	14.20	/	/
D_9_-1-Hydroxypyrene	D_9_-1-OHPyr	299.1692	20.08	/	/
^13^C_12_-2，3，3′，4，4′-Pentachlorobiphenyl	^13^C_12_-PCB-105	339.9178	17.65	/	/

为了减少个体差异、饮食习惯、水分摄入等因素对尿中OH-PAHs检出含量的影响，本研究采用尿肌酐校正尿液OH-PAHs含量。尿肌酐采用高效液相色谱法进行测定^［14］^。

### 1.4 质量保证

为确保结果的准确性和可靠性，采取了质量控制和保证措施^［15］^。每次分析运行都包括8个标准曲线点、一个程序空白和两个标准参考物质（NIST SRM 3672），以确保分析的稳定性和灵敏度，并监测潜在的污染。所有目标分析物均采用内标法定量。所有目标分析物标准曲线的相关系数（*r* ^2^）>0.99。每批样品回收率内标的相对标准偏差不大于10%。NIST SRM 3672的检测值均在参考值范围内。此外，OH-PAHs和尿肌酐测定均通过德国外部质量评估计划（G-EQUAS）考核。

### 1.5 统计分析

低于检出限的检测结果用检出限的1/2代替。与每种母体化合物相关的OH-PAHs含量合并后作为代谢物的总含量进行分析。具体来说，1-OHNap和2-OHNap合并为OHNap；2-OHFlu、3-OHFlu和9-OHFlu合并为OHFlu；1-OHPhe、2-OHPhe、3-OHPhe和4-OHPhe合并为OHPhe；羟基芘则仅有1-OHPyr；以上10种OH-PAHs合并为OH-PAHs的总和（∑OH-PAHs）。通过Spearman秩相关分析（双尾）评估OH-PAHs间的相关性；比较各人口组代谢物含量的分布情况时，使用非参数Mann-Whitney U和Kruskal-Wallis H检验。在所有分析中，*p*<0.05视为有统计学意义。

## 2 结果与讨论

### 2.1 尿液样本中OH-PAHs的检出率和含量分布

北京居民尿液样本中OH-PAHs的含量用ng/g表示，结果见表3。1-OHNap、2-OHNap、2-OHFlu、9-OHFlu、1-OHPhe和2-OHPhe等6种OH-PAHs的检出率达到100%，3-OHFlu、3-OHPhe和4-OHPhe、1-OHPyr的检出率分别高达99%和91%。这表明PAHs暴露在北京地区的人群中非常普遍。

**表 3:** 92份尿样中10种OH-PAHs的检测结果

Compound	Detection rate/%	Content/（ng/g）	GM/ （ng/g）	Percentiles/（ng/g）					
				P_10_	P_25_	P_50_	P_75_	P_90_	IQR
1-OHNap	100	63‒14589	556	152	248	539	1086	2562	838
2-OHNap	100	146‒15185	858	252	414	735	1366	3503	952
2-OHFlu	100	43‒1236	144	61	76	132	199	486	123
3-OHFlu	99	1‒945	50	18	27	40	79	222	51
9-OHFlu	100	30‒4214	291	57	94	299	860	1645	766
1-OHPhe	100	19‒453	67	37	42	63	89	172	47
2-OHPhe	100	21‒630	67	33	44	59	92	177	48
3-OHPhe	99	2‒1386	56	13	25	54	133	280	108
4-OHPhe	91	1‒247	25	11	19	28	43	69	24
1-OHPyr	91	1‒1077	41	9	24	43	76	177	52
OHNap	100	231‒29773	1502	570	735	1320	2225	6560	1489
OHFlu	100	135‒4426	616	212	291	506	1337	2305	1046
OHPhe	100	51‒1668	257	126	164	228	395	584	231
ΣOH-PAHs	100	661‒33782	2775	1173	1582	2431	3665	8481	2083

GM： geometric mean； IQR： interquartile range. OHNap： 1-OHNap+2-OHNap； OHFlu： 2-OHFlu+3-OHFlu+9-OHFlu； OHPhe： 1-OHPhe+2-OHPhe+3-OHPhe+4-OHPhe； ΣOH-PAHs： total of the ten OH-PAHs.

尿液样本中ΣOH-PAHs的含量范围为661～33 782 ng/g，总体几何平均值（GM）为2 775 ng/g，揭示了个体间PAHs暴露水平存在显著差异。在所有检测的OH-PAHs中，OHNap的含量最高，占ΣOH-PAHs的62.2%，其次是OHFlu和OHPhe，而1-OHPyr的含量最低。这种含量分布模式（OHNap>OHFlu>OHPhe>1-OHPyr）与全球其他地区的研究结果相一致^［16］^，强调了OHNap作为评估PAHs暴露生物标志物的重要性。OH-PAHs的含量与分子大小之间存在显著负相关，表明分子的大小会影响吸收效率、生物利用率、代谢途径和生物效应，对PAHs在体内的吸收、分布、代谢和排泄有决定性影响。在排泄过程中，较小的PAHs（即具有2～3个芳香环）的代谢物倾向于通过尿液排泄，而较大的PAHs的代谢物则主要通过粪便排泄^［17］^。值得注意的是，虽然某些OH-PAHs的含量较低，如OHFlu、OHPhe和1-OHPyr，但它们具有致癌性和致突变性，即使在低剂量暴露下也可能对人体健康构成潜在风险^［18］^。这些数据为评估不同OH-PAHs的暴露风险提供了重要依据。

### 2.2 尿液中OH-PAHs含量的相关性

通过Spearman秩相关分析（见图1），我们发现多对OH-PAHs之间存在显著的正相关关系，如1-OHNap与2-OHNap、2-OHFlu、3-OHFlu及1-OHPhe，这些强正相关表明它们可能具有相似的暴露源和生物代谢过程。尤为值得注意的是，9-OHFlu展现出与众不同的相关性模式，与其他OH-PAHs呈现负相关或弱相关关系，这一现象引发了对其潜在独特暴露源或生物转化路径的深入思考。与其他研究中2-OHFlu与3-OHFlu含量占主导地位的结论不同，本研究中9-OHFlu的含量显著高于2-OHFlu和3-OHFlu。我们认为9-OHFlu可能来源于除了直接暴露于PAHs之外的其他途径，如医药、农业及染料工业中广泛使用的芴酮类化合物，这些化合物在人体或环境中的代谢转化可能是尿液中9-OHFlu高含量水平的一个贡献因素^［19］^。因此，对9-OHFlu的监测与评估需要纳入更广泛的污染源考量，以全面理解其环境与健康影响。

**图 1:**
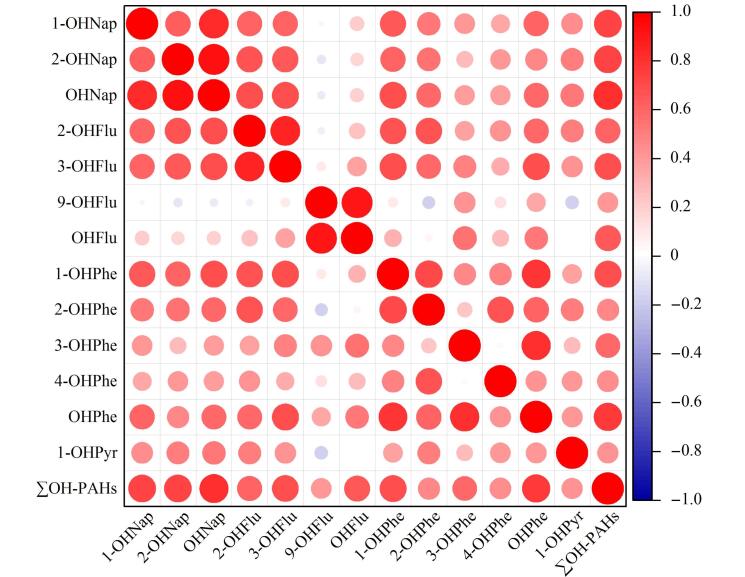
尿样中OH-PAHs相关性热图

### 2.3 年龄、性别、吸烟习惯与OH-PAHs含量水平的关系

将研究对象按年龄、性别、吸烟习惯等级分组，并进行统计分析，不同组别中OH-PAHs的含量水平如图2所示。

**图 2:**
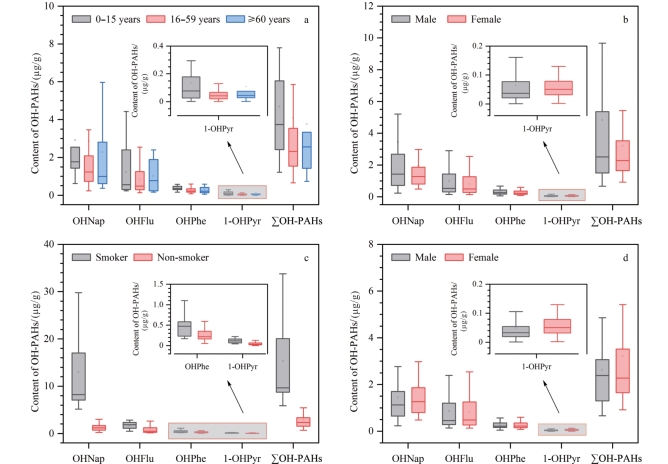
不同（a）年龄、（b）性别、（c）吸烟习惯及（d）排除吸烟影响后的性别分组中OH-PAHs的含量

研究结果显示，年轻群体（尤其是0～15岁），OHNap的含量水平显著高于其他年龄组（图2a），这可能与该年龄段特有的活动模式，如户外游戏、接触土壤及可能的高污染环境暴露源（家庭装修材料释放）等有关。随着年龄增长，OHPhe的含量水平逐渐下降，可能与个体行为模式的改变、居住环境质量的提升以及体内代谢能力的变化有关。值得注意的是，OHFlu和1-OHPyr在老年组（≥60岁）中的含量水平略有上升，这揭示了随着年龄增长，代谢途径或暴露源可能发生了特异性变化，如药物使用、饮食习惯或居住环境的不同，这些因素共同作用于OH-PAHs的体内代谢与排泄过程。∑OH-PAHs作为PAHs暴露综合指标，其含量水平在少年儿童组（0～15岁）达到峰值（GM为3 940 ng/g），随后在劳动年龄组（16～59岁，GM为2 598 ng/g）及老年组（≥60岁，GM为2 639 ng/g）中趋于平稳且水平相近，这一趋势进一步强化了年龄作为OH-PAHs暴露模式重要影响因素的观点。此外，各年龄段OH-PAHs的暴露数据的右偏分布特征，揭示了暴露水平的非均匀性，即少数个体面临较高的暴露风险，而大多数人群处于相对较低水平。

性别差异在PAHs暴露中同样显著且复杂（图2b）。男性的OHNap含量水平不仅整体高于女性，还表现出更高的变异性，这可能与男性更高的户外活动频率及吸烟习惯等因素有关。相比之下，OHFlu的含量分布较为集中，且女性群体的含量水平低于男性，这可能反映了性别间在环境暴露源接触上的差异。对于OHPhe与1-OHPyr，尽管两性均处于较低暴露水平，但男性OHPhe略高，女性1-OHPyr略高，尽管这些差异在统计学上并不显著（*p*>0.05），但仍提示了性别间代谢及行为模式的微妙差异。

吸烟习惯对OH-PAHs含量有一致且显著的影响，吸烟者的所有代谢物含量都高于非吸烟者（图2c）。吸烟不仅增加了吸烟者直接吸入PAHs的风险，同时也通过影响体内代谢过程增加了OH-PAHs的含量。这些发现为理解吸烟在PAHs暴露中的作用提供了科学依据，并为制定公共卫生干预措施提供了重要信息。值得注意的是，在排除吸烟等潜在干扰因素后（图2d），女性1-OHPyr的含量显著高于男性（*p*=0.03），这一发现与先前研究相一致^［20］^。且有研究^［21］^表明，PAHs摄入量较低的女性受试者尿液中OH-PAHs含量高于男性，进一步强调了性别间代谢差异及生活习惯对PAHs暴露的潜在影响。这一发现不仅丰富了我们对OH-PAHs暴露机制的理解，也为制定性别特异性的健康干预策略提供了科学依据。

## 3 结论

本研究采用先进的气相色谱-高分辨双聚焦磁质谱联用技术，结合精确的样品预处理方法，对北京居民尿液中10种关键OH-PAHs进行了定量分析。该技术具有高灵敏度和高特异性，能够准确反映人体对PAHs的暴露水平，为流行病学研究和健康风险评估提供了可靠的基础数据。随着城市化进程的加快，对于北京这样的人口密集型大都市，面临着PAHs污染的严峻挑战，OH-PAHs作为PAHs暴露的生物标志物，其含量水平与人体健康风险密切相关。本研究揭示了北京居民PAHs暴露的普遍性及个体间差异性，强调了OHNap作为主要暴露标志物的重要性，并观察到OH-PAHs含量与分子大小之间的负相关性，为深入理解PAHs在人体内的动态变化提供了新的视角。本研究通过Spearman秩相关分析，揭示了9-OHFlu的独特相关性模式，为探索不同化合物的暴露源和生物代谢过程提供了新的视角。此外，本研究还探讨了年龄、性别和吸烟习惯对OH-PAHs含量的影响，发现这些人口统计学因素在PAHs暴露中的显著作用，为制定针对性的健康干预措施提供了科学依据。这些发现对于优化环境保护策略、制定公共卫生干预措施，尤其是针对易感群体，具有重要的指导意义，并对于理解和应对城市化进程中的环境健康挑战具有重要的学术价值和实际应用价值。

本研究样本量有限，未能为北京居民尿液中OH-PAHs的含量水平提供全面的参考值，建议后续研究在本研究基础上扩大样本量，进行更深入的分析。未来研究应聚焦暴露源、环境介质与人群特征的综合作用，持续监测PAHs代谢物，并深入探讨其分子结构和大小与健康效应间的复杂关系，以全面评估PAHs污染的健康风险。
